# Dendroclimatological study of ancient trees integrating non-destructive techniques

**DOI:** 10.3389/fpls.2024.1469675

**Published:** 2024-10-09

**Authors:** Jinkuan Li, Yameng Liu, Yafei Wei, Jiaxin Li, Keyu Zhang, Xiaoxu Wei, Jianfeng Peng

**Affiliations:** ^1^ College of Geography and Environmental Science, Henan University, Kaifeng, China; ^2^ The Key Laboratory of Earth System Observation and Simulation of Henan Province, Kaifeng, China

**Keywords:** ancient trees, non-destructive technology, peak-valley analysis, tree ring index, climate response

## Abstract

Based on the need to protect previous ancient trees and the development of dendroclimatology, the use of non-destructive technologies in tree-ring research has gained increasing attention. This study focuses on the ancient *Pinus tabulaeformis* in Yu Xiang Forest Farm in Henan Province. Firstly, samples were collected using the traditional Increment borers and the Resistograph, a non-destructive method. Subsequently, the peak-valley analysis was used to filter the data obtained by the Resistograph to extract the tree ring width sequence, and the data’s accuracy was verified by correlation analysis with tree ring width sequence by the Increment borers. Then, the optimal filtering method and an appropriate comprehensive threshold were determined, and tree ring width and density sequences were successfully extracted. Following that, the growth trend and residual resistance in the measurement process were corrected using linear fitting and Ensemble Empirical Mode Decomposition (EEMD) technology, thereby establishing the tree-ring width and density index series, which were further validated through correlation analysis and t-tests. Finally, analysis of the correlation with climatic factors, identified the main limiting factors for tree growth, and the accuracy of the tree-ring information extracted by the Resistograph was further verified. The results showed that spite of certain differences between the tree-ring width indices extracted by the Resistograph and the Increment borer, they were generally reliable. The radial growth of the ancient *P.tabulaeformis* in Yu Xiang Forest Farm is primarily influenced by temperature, with the maximum density of the tree rings responding more significantly to the mean maximum temperature, while the minimum density of the tree rings responded more significantly to the mean minimum temperature. These results not only provide a scientific and accurate age for the protection of ancient trees and verify the reliability of the data obtained by the Resistograph, but also facilitate the use of non-destructive technology for in-depth study of ancient trees, therefore enhancing our understanding of how climate change affects tree growth and provide valuable insights for the future protection and management of these ancient trees.

## 1 Introduction

Global warming has become an undeniable fact (IPCC, 2021). With the warming of the global climate, the growth environment of trees faces huge challenges. In recent years, climate warming has led to increasing drought events and subsequent forest decline or death (Peng et al., 2011; Gauthier et al., 2015; Millar and Stephenson, 2015). As an important part of nature, tree growth not only is related to ecological balance, but also directly affects the living environment of human beings. Ancient trees play a crucial role in maintaining biodiversity, ecological balance, carbon storage, soil and water conservation, microclimate regulation, and providing aesthetic and cultural value (Seibold et al., 2018; Wu, 2019). They are indispensable components of ecosystems, providing habitats for many species and serving as important resources for scientific research and education (Takács and Malatinszky, 2021). Protecting ancient trees helps ensure the long-term preservation and utilization of these valuable resources (Gao, 2023; Ren et al., 2024).

Traditional tree ring research has some limitations in exploring environmental information of ancient trees because the protection and possible influence of ancient trees should be considered in sample collection (Xu et al., 2012). With the development of science and technology, a series of innovations have occurred in sampling technology, from the traditional Increment borers sampling to X-ray (Polge, 1966; Parker and Meleskie, 1970), stress wave (Bulleit and Falk, 1985; Schad et al., 1996), ultrasonic detection (Bauer et al., 1991; Yang and Wang, 2010), and Resistograph (Wang et al., 2013; Xia, 2023), and other non-destructive non-sampling detection equipment. These innovations, therefore, greatly improve the efficiency and convenience of data collection. However, most of the equipment is bulky and inconvenient to carry and use in the field. In contrast, the Resistograph is easy to carry, simple to use, low cost, and widely applicable, thus becoming an economical and efficient non-destructive tree ring measurement tool (Wang et al., 2013; Xia, 2023). Moreover it has gradually been applied in the analyses of tree rings (Rinn et al., 1996; Chantre and Rozenberg, 1997; Wang and Lin, 2001; Lima et al., 2007; Acuna et al., 2011; Liang, 2017; Wei, 2018; Pan, 2020; Xia, 2023; Zhang, 2024), tree decay detections (Rinn, 1994; Costello and Quarles, 1999; Ceraldi et al., 2001; Wu, 2011a; Wu et al., 2011b; Zhu et al., 2013; Nutto and Biechele, 2015), and the assessment of wood structure status (Winistorfer et al., 1995; Isik and Li, 2003; Zhang et al., 2007; Ukrainetz and O’Neill, 2010; Sun et al., 2011; Sun, 2012) and other fields. Using the Resistograph identification method, we can more comprehensively understand the history and growth of ancient trees, providing a scientific basis for their protection and inheritance. However, the issue of dating accuracy has not yet been resolved. The application of the Resistograph, both domestically and internationally, has not been widely popularized, especially in tree ring width and density research.

Henan Province, located in the central part of China, is crossed by the Qin-Huai line in its southern region and mostly features a warm temperate continental monsoon climate (Wang et al., 1990). The natural geographical environment is complex and diverse, and the historical and cultural heritage is profound, with a wealth of ancient tree resources. As a key area for ecological protection and high-quality development in the Yellow River basin, the ancient tree resources of Henan Province are of great significance to the construction of ecological civilization and the inheritance of historical culture (Ren et al., 2024). Therefore, it is critical/important to use ancient trees to study long-term climate change in this region for clarifying the evolution of civilization in the Central Plains.

The objectives of this study were to: 1) determine the optimal filtering method and the best comprehensive threshold for extracting tree ring information from ancient trees using the Resistograph; 2) establish chronologies of tree ring width and density (mean density, minimum density, and maximum density) indices; 3) identify and analyze the main climatic limiting factors that affect tree growth; 4) ascertain the reliability of the Resistograph sequence and the feasibility of ancient tree-ring research. The study also aimed to provide a new perspective for the extraction of tree ring information using the Resistograph and to offer a scientific basis for the protection and management of ancient trees.

## 2 Materials and methods

### 2.1 The study area

**Figure 1:**
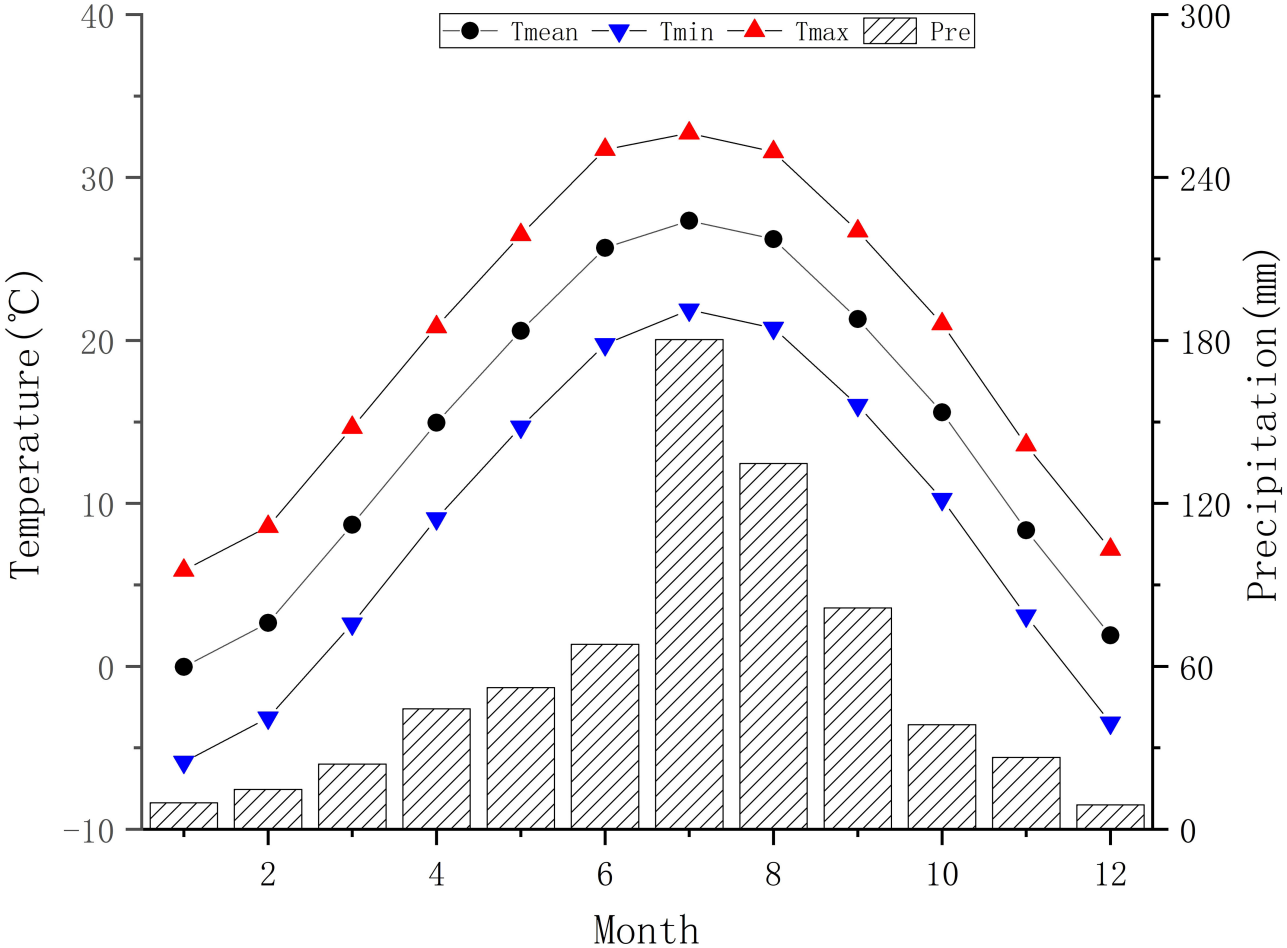
Gridded monthly meteorological data statistical chart for sampling in Yu Xiang Forest Farm.

Yuxiang Forest Farm (34°28’-34°29’N, 114°56’-114°57’E) is located in Suixian County, Shangqiu City, Henan Province, covering an area of nearly 70 km^2^.The area is situated in the eastern plains of Henan Province, within the Yellow River alluvial fan region, with flat terrain and an elevation ranging from 51 to 60 meters. Climatically, Yuxiang Forest Farm has a warm temperate semi-humid continental monsoon climate with mildly wet summers and cold dry winters (Zhang et al., 2023; Ji, 2022), as depicted in **Figure 1**. It has moderate and evenly distributed precipitation, providing favorable conditions for vegetation growth (Li and Jing, 1999). Despite the frequent human activity, low altitude, and relatively infertile soil conditions, the forest still stands tall with 96 ancient *Pinus tabuliformis*, each measuring 40 centimeters in diameter and nearing a century in age. These ancient trees have been officially recorded as part of the *P.tabuliformis* ancient tree group during the national survey of ancient tree clusters. They not only demonstrate remarkable survival capabilities and adaptability to their environment, but also have distinct annual rings, making them a valuable resource for dendroclimatic research.

### 2.2 Research methods

#### 2.2.1 Introduction to the Resistograph

**Figure 2:**
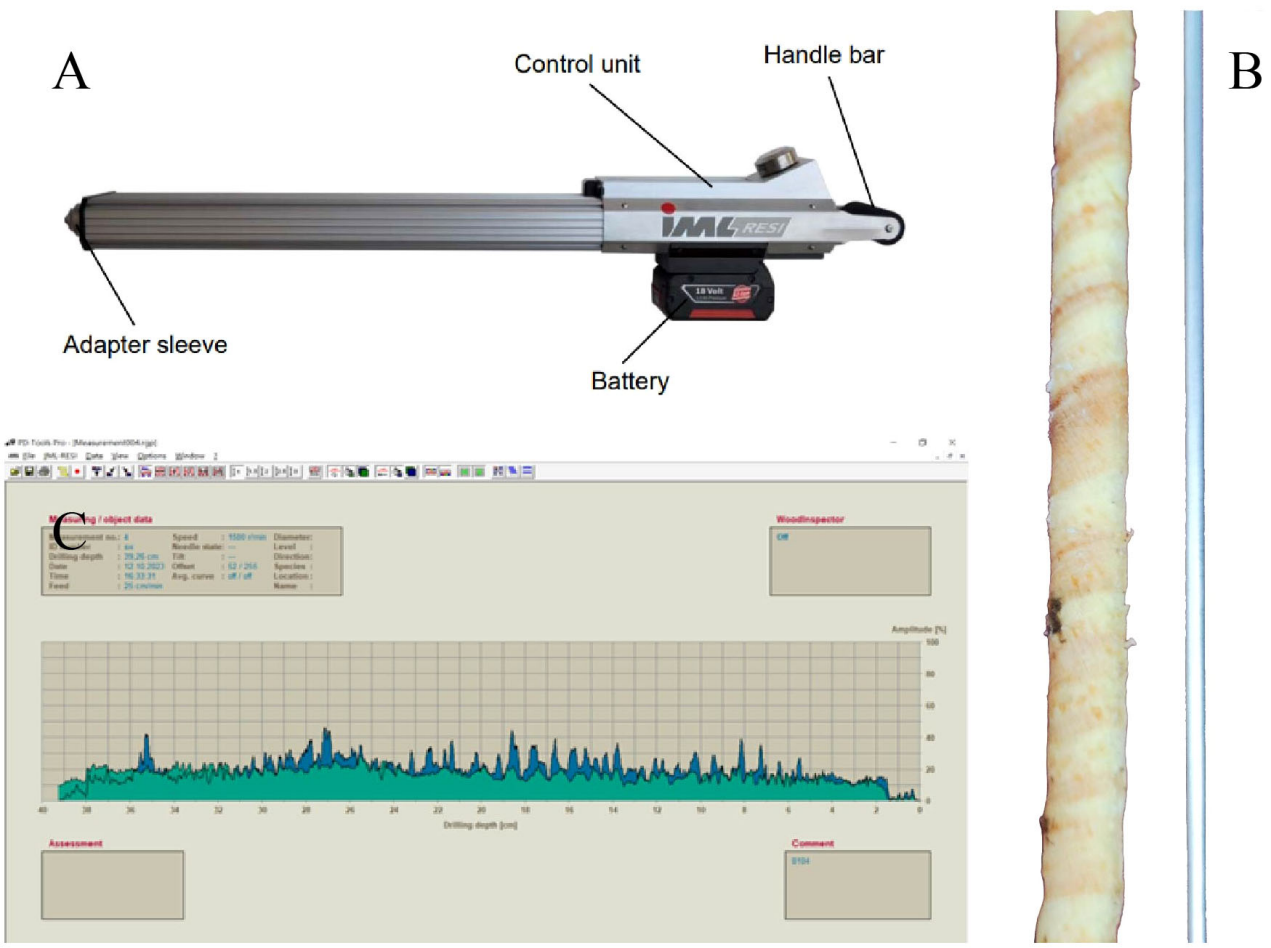
Resistograph and its application [**(A)**: Picture of the Resistograph; **(B)** Size comparison between the steel needle of the Resistograph (diameter 1.5mm) and the Increment borers sample embedded in the wooden slot (diameter 5.15mm); **(C)** Resistance curve exported by the Resistograph, green represents DR, blue represents FR, with a data resolution of 1/10 mm].

The Resistograph used in this study is the Germany-made Resistograph PD600 model (**Figure 2A**). The probe needle of this device is made of special steel with a diameter of only 1.5mm (**Figure 2B**), capable of measuring trees with a diameter of up to 200cm. The resistance curve diagram generated can provide key data such as drilling resistance (DR, the resistance encountered by the drill bit during rotation), feed resistance (FR, the resistance encountered by the drill bit during advancement), and core information (**Figure 2C**). These curve diagrams can not only intuitively display the internal structure of the trees but also identify problems such as decay or cavities. Data are stored in the built-in microcomputer of the device and can be further processed and analyzed through the PD-Tools Pro software.

Detection Principle: The Resistograph is based on the resistance changes when the probe drills into the xylem, enabling it to infer wood characteristics such as age, tree ring width, and density. The advantages include rapid and accurate detection capabilities, simple operation, and non-destructive in nature, making it suitable for dendroclimatic studies of ancient trees (Yao et al., 2022a; b; 2023).

#### 2.2.2 Samples collection

In October 2023, samples were collected from Yuxiang Forest Farm. The study adhered to the standards of the International Tree-Ring Data Bank (ITRDB), selecting thicker trees for sampling based on the sensitivity and replication principles of the trees. With an Increment borer, 2 cores were drilled from the base of each tree. To prevent sample damage, the samples were quickly placed into test tubes for preservation and numbered, for a total of 20 trees and 40 cores. Subsequently, in order to facilitate comparison with the Increment borer samples, the Resistograph was used to align the probe 3 cm above or 3 cm below the Increment borers sampling point. After starting the device, the probe was steadily inserted into the tree, recording the resistance changes and exporting the resistance curve from bark-pith-bark. It is necessary to keep the device stable during the measurement, and after the measurement, the data were then brought back to the laboratory for analysis.

#### 2.2.3 Tree-ring information based on the increment borers

In the laboratory, all core samples were fixed, air-dried, sanded, measured for width, and cross-dated according to the methods of Stokes ma Smiley-TL (1968) and others in the ITRDB. The cross-dating results were corrected using the COFECHA program (Holmes, 1983), and any missing or false tree rings in the samples were corrected and quality controlled. Samples with low consistency with the main sequence were excluded. Finally, 20 trees with 40 core samples were retained. The age and ring width of each core sample were determined.

#### 2.2.4 Extraction and verification of tree-ring information based on the Resistograph

In the laboratory, the data from the Resistograph were processed using Excel software to eliminate errors that might be caused by the drill bit entering and exiting the tree, and to exclude the potential impact of bark thickness on the measurement results, thus reducing the overall error range. The density differences between the earlywood (EW) and latewood (LW) of the tree cause resistance values to fluctuate (Yao et al., 2022c). The resistance line graph illustrates these variations with multiple peaks and valleys. However, not all peaks and valleys represent real tree ring changes; some minor fluctuations are indicative of false rings (Yao et al., 2022d; Pan, 2020). Therefore, setting an appropriate resistance threshold (Det) is necessary to distinguish between real tree rings and false rings.

The specific process for extracting the tree ring width sequence is as follows:

(1) Code the data from the Resistograph;
(2) Extract all extreme points;
(3) Filter the Resistograph data using several different methods of resistance threshold (Det) to remove minor peaks and valleys;
(4) Extract the tree ring width sequence using valid peaks and valleys (the encoding difference between extremes is directly related to the measurement of tree ring width, considering that the measurement accuracy of the Resistograph PD600 is 0.1mm).

##### 2.2.4.1 Selection of the optimal filtering method

Based on past research and tree growth trends, eight threshold methods were selected to filter the resistance curve, making the age extracted by the Resistograph closest to the age extracted by the increment borers. The methods are as follows:

- Fixed threshold;
- Linear threshold starting at 0 (assign the value for the Resistograph near the bark as 2022, and the innermost value as the starting year determined by the Increment borers, with linear interpolation in between. Based on the growth trend of the tree ring width extracted by the Increment borers, a linear threshold is set. If the growth trend of the tree ring width extracted by the Increment borers continues to decline and the fitting curve is always greater than 0, then the threshold for the Resistograph to extract the tree ring width is set as a linear threshold that continues to decline to 0; if the growth trend of the tree ring width extracted by the Increment borers continues to decline and fits to 0 after a certain number of years, then a linear threshold that declines and the fitting curve is greater than 0 is set for the years where the growth trend declines and the fitting curve is greater than 0, and a threshold of 0 is set for the years where the fitting curve is less than 0);
- Linear threshold starting at 0.01;
- Linear threshold starting at 0.02;
- Linear threshold starting at 0.03;
- Linear threshold starting at 1/10 of the maximum threshold value in method B;
- Linear threshold starting at 2/10 of the maximum threshold value in method B;
- Linear threshold starting at 3/10 of the maximum threshold value in method B.

The tree ring width sequences extracted by the Resistograph using the eight methods were analyzed for correlation with the tree ring width sequences extracted by the Increment borers to determine the optimal filtering method.

##### 2.2.4.2 Selection of the optimal comprehensive threshold

**Figure 3:**
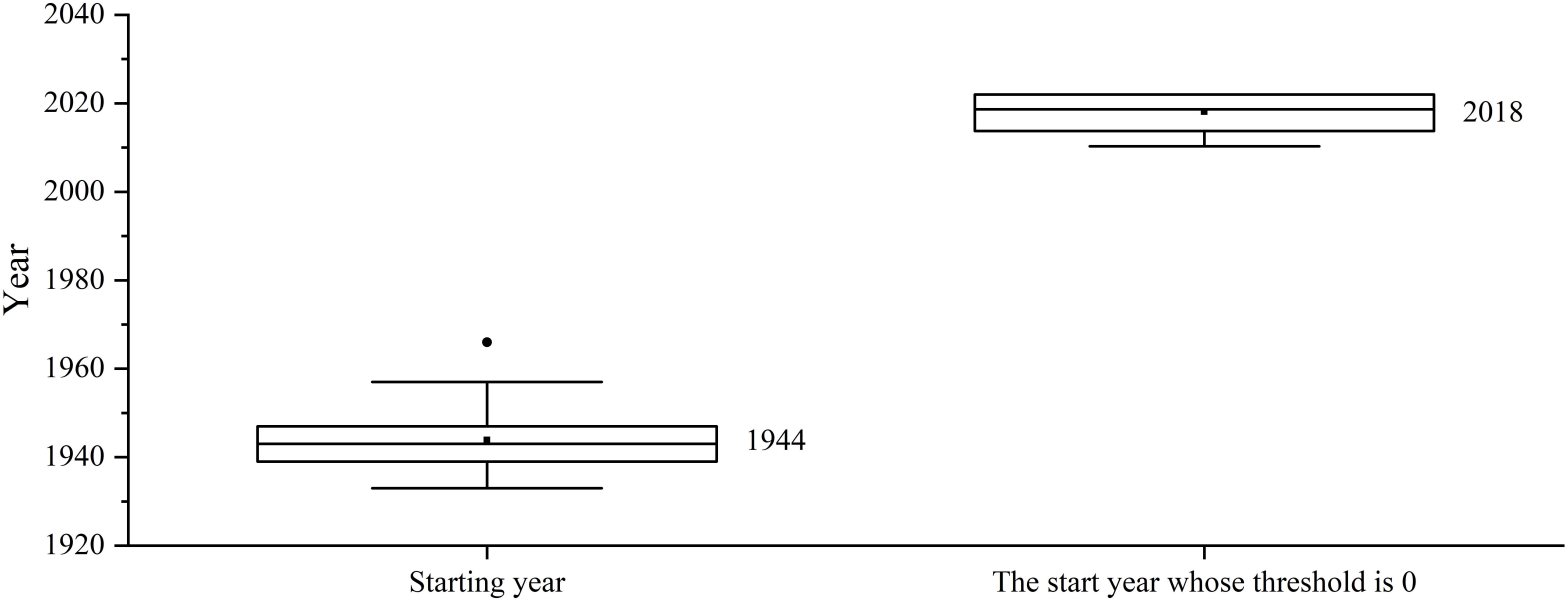
The mean start year of regional tree growth compared to the start year with an initial threshold of 0.

The Resistograph assigns the value near the bark 2022 year, and the innermost value as the comprehensive starting year of the tree ring data extracted by the Increment borers (1944, **Figure 3**), with linear interpolation in between. The overall growth trend of the trees in this area continues to decline, fitting to 0 in the year 2018 (**Figure 3**), and the maximum threshold is determined based on the correlation between tree ring width sequence extracted by the Increment borers and the corresponding tree ring width sequence extracted by the Resistograph, with linear interpolation in between.

#### 2.2.5 Tree-ring data standardization

The variation in tree ring width is influenced by genetic and environmental factors. Genetics mainly affect the ring width through tree age; as the tree ages, the ring width changes, a phenomenon known as “growth trend”. When measuring with a Resistograph, the probe penetrates the tree rings, increasing the contact area and friction, which leads to measured values that include a combination of density and friction, with the friction part being residual resistance.

To improve the accuracy of ring width and density measurements, linear fitting and Ensemble Empirical Mode Decomposition (EEMD) are used to remove signals caused by the tree’s own characteristics and non-synchronous microhabitat factors (Fritts, 1976), and residual resistance during density measurement (Xia, 2023). This helps accurately analyze and interpret tree ring data, and better understand the relationship between tree growth and climate change.

The principle of standardization: according to the fitted curves of tree width and density, the expected growth value for the tree species is calculated, reflecting the degree to which the ring is influenced by meteorological changes after excluding other influences. The formula is:


It=Wt/Yt

where: *It* is the ring index of ring width (density) in year *t*; *Wt* is the measured value of ring width (density) in year *t*; *Yt* is the expected value of ring width (density).

#### 2.2.6 Acquisition of meteorological data

To address the differences in altitude and distance between the sampling site and the meteorological station, this study used the 1km resolution monthly meteorological dataset provided by the Qinghai-Tibet Plateau Data Center (Peng, 2020a; b; c; d) to extract monthly mean temperature (Tmean), mean minimum temperature (Tmin), mean maximum temperature (Tmax), and precipitation (Pre) near Yuxiang Forest Farm (34°28’47”-34°29’17”N, 114°56’31”-114°57’1”E) from 1950 to 2021 (**Figure 1**). Obviously, this area belongs to a continental monsoon climate, characterized by precipitation concentrated in July-August and concurrent precipitation and heat during the monsoon season. The mean annual Tmean is 14.44°C and mean annual precipitation is 683.57mm.

#### 2.2.7 Correlation analyses

The DendroClim2002 (Biondi and Waikul, 2004) was used to analyze the correlation between the tree ring indices and climatic factors. The aim was to explore the response of tree growth to climate change and determine the main climatic factors limiting tree growth. Tree ring indices included ring width indices measured by Increment borers, as well as, ring width and density indices (mean, minimum, maximum) extracted from Resistograph curves. Considering the possible “lag effect” of climatic factors on tree growth, this study selected 21 months of climatic data from the previous March to the current November for analysis.

## 3 Results and analysis

### 3.1 Results of different filtering methods

The optimal filtering method was selected by comparing eight methods (**Figure 3**) through correlation analysis between the tree ring width sequences extracted by the Resistograph and the sequences measured by the Increment borers.

**Figure 4:**
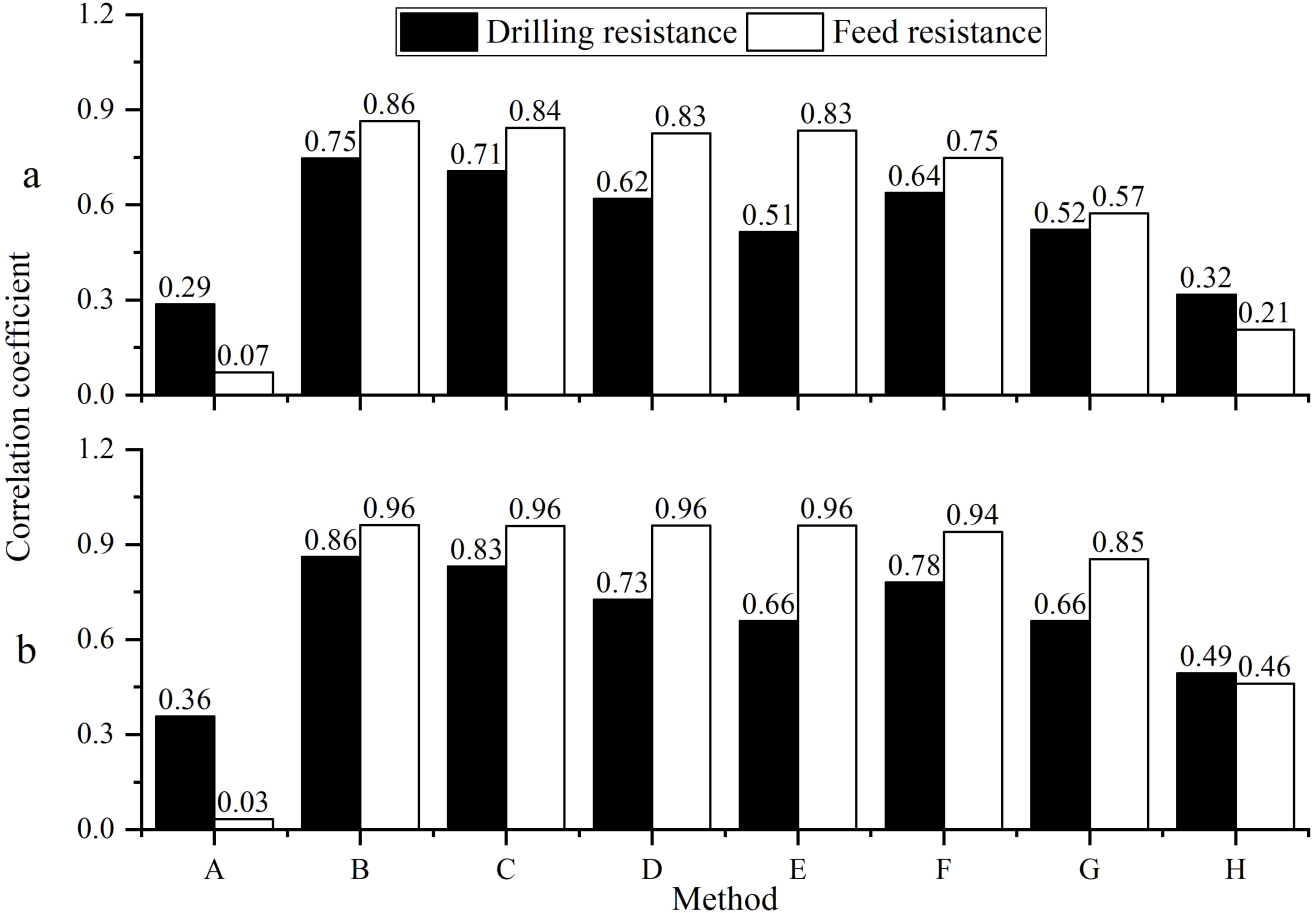
The correlation of the comprehensive tree ring width sequence **(A)** was extracted using both the Resistograph and the Increment borers, their 5-year moving averages **(B)**.

The correlation analysis (**Table 1**) revealed that there are a higher number of significant correlations between tree ring width sequences extracted by the Resistograph (A linear threshold whose initial threshold is 0, **Table 1** B column) and the Increment borers, with the best correlation observed between the comprehensive sequences (**Figure 4**), indicating that the tree ring width sequence extracted by the Resistograph using an initial threshold value of 0 for the linear threshold is credible for both high-frequency (**Figure 4A**) and low-frequency signals (**Figure 4B**).

**Table 1:** Number of significant correlations in each method (The tree ring width sequences, including both the individual sequences extracted using the Resistograph and the Increment borers (I), their 5-year moving averages (II), the individual sequence extracted using the Resistograph and the comprehensive tree ring width sequence derived from the mean of all sequences using the Increment borers (III), their 5-year moving averages (IV)), the same below.

		A	B	C	D	E	F	G	H
I	Drilling Resistance	18	44	41	30	27	33	23	19
	Feed Resistance	3	43	38	37	37	32	13	10
II	Drilling Resistance	36	52	52	51	49	52	42	40
	Feed Resistance	19	52	52	51	51	49	31	17
III	Drilling Resistance	13	30	30	25	23	25	18	17
	Feed Resistance	7	30	29	26	26	24	13	8
IV	Drilling Resistance	22	31	31	31	29	31	27	24
	Feed Resistance	13	31	31	30	30	30	28	15

### 3.2 Optimal comprehensive threshold

**Figure 5:**
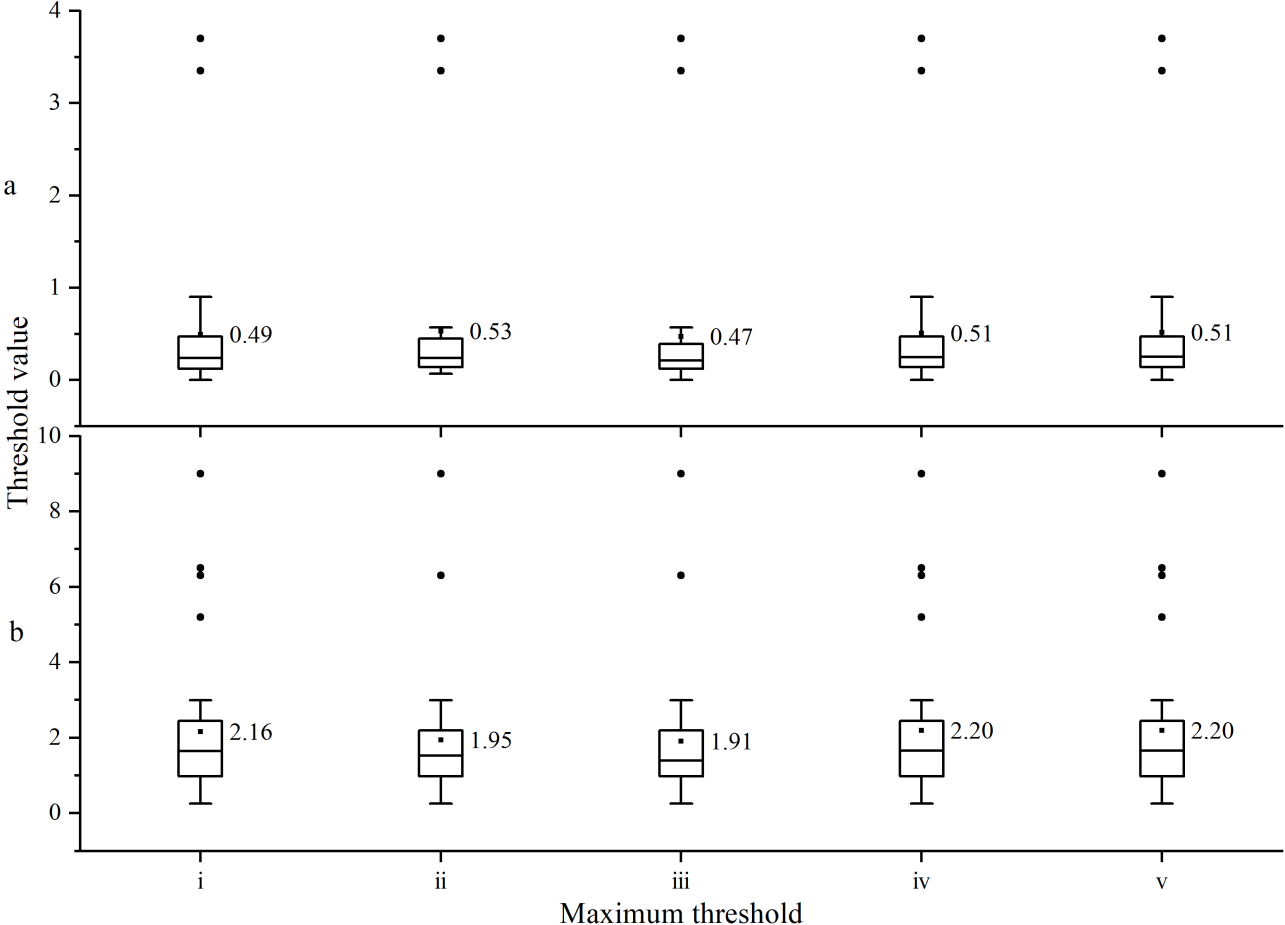
Maximum threshold values of **(A)** drilling resistance and **(B)** feed resistance.

Using an initial threshold value of 0(2018-2022, **Figure 2**), the maximum threshold values as shown **Figure 5**(i: The mean of all maximum threshold values; ii: iii: iv: v: The average values of the maximum thresholds for significant correlations in sequences I, II, III, and IV after removing outliers), for the linear threshold values in between, linear interpolation is applied.

Using the correlation analysis of the tree ring width sequences extracted by the Resistograph and the Increment borers, the optimal comprehensive threshold is selected from among five candidate thresholds.

**Figure 6:**
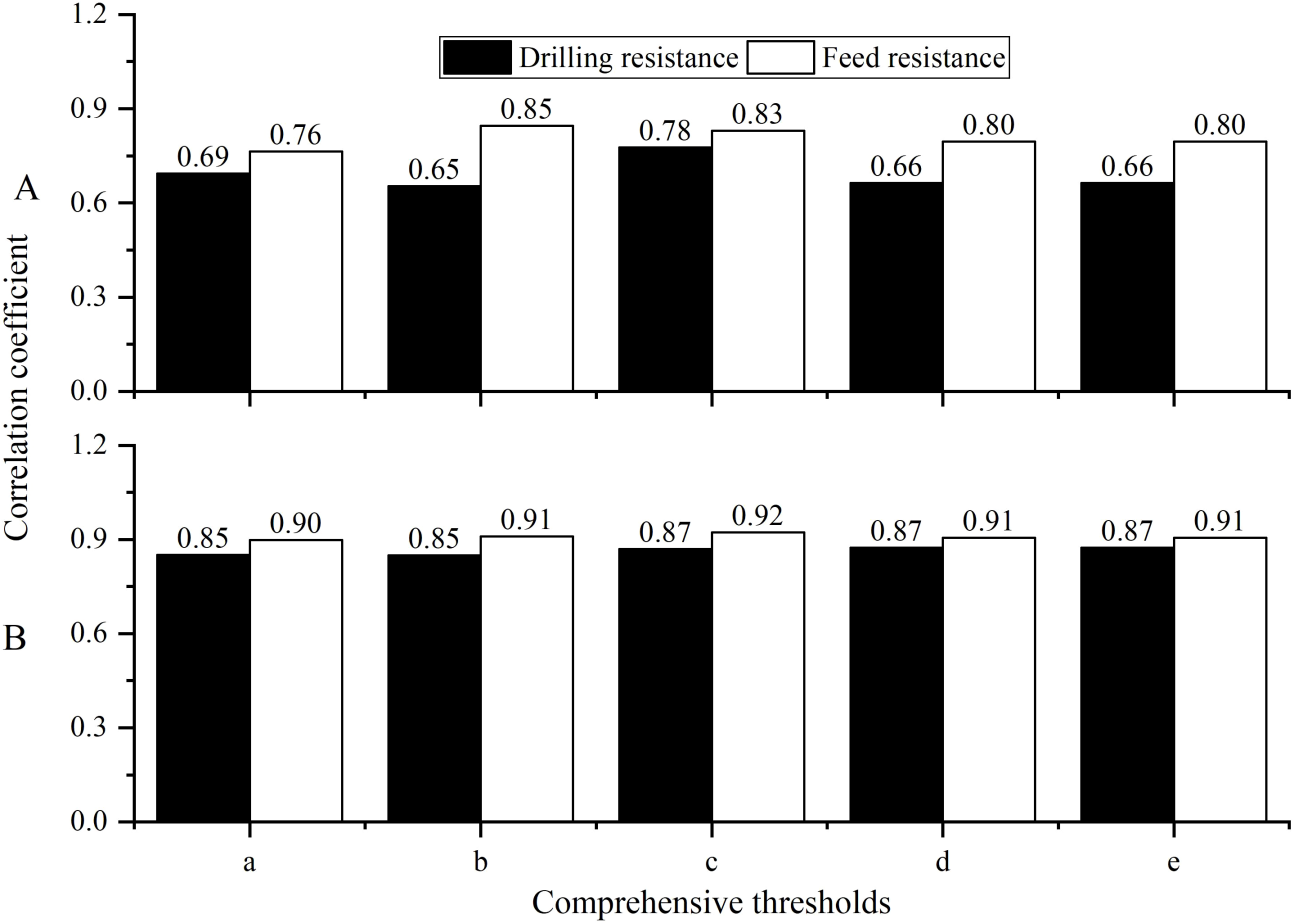
The correlation of the comprehensive tree ring width sequence **(A)** was extracted using both the Resistograph and the Increment borers, their 5-year moving averages **(B)**.

Through correlation analysis, it was found that the tree ring width sequences, extracted using the Resistograph with five different methods and the Increment borers, have a higher number of significant correlations (**Table 2**), with the best correlation observed between the comprehensive sequences (**Figure 6**). However, method c is the best. This indicates that the tree ring width sequences extracted by the Resistograph using the comprehensive threshold value are credible for both high-frequency (**Figure 6A**) and low-frequency (**Figure 6B**) signals.

**Table 2:** Number of significant correlations in each comprehensive threshold.

		i	ii	iii	iv	v
III	drilling Resistance	39	40	39	39	39
	Feed Resistance	38	38	38	38	38
IV	drilling Resistance	41	41	41	41	41
	Feed Resistance	41	41	41	41	41

### 3.3 Establishment of tree ring indices

#### 3.3.1 Tree ring width index

**Figure 7:**
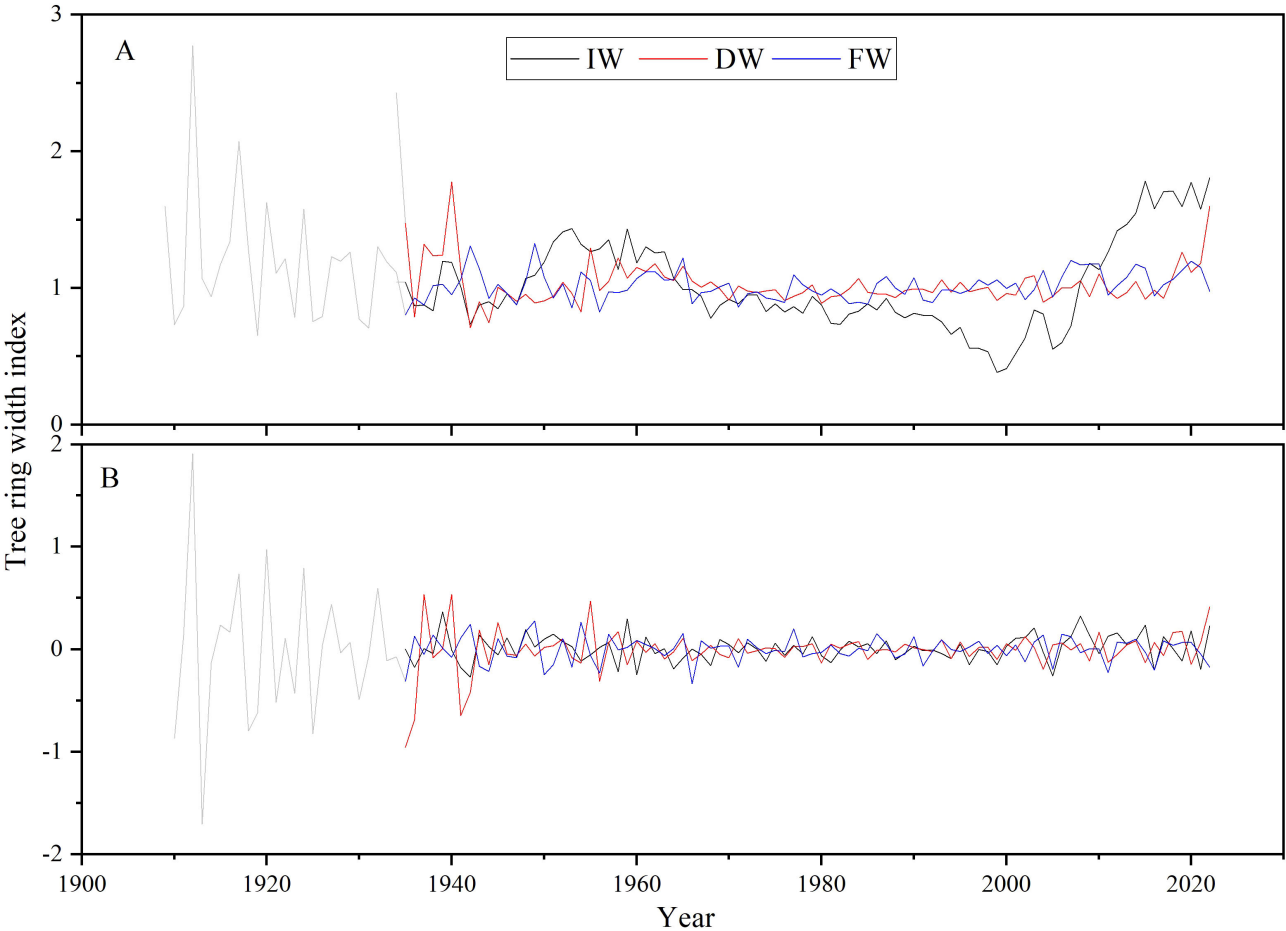
Tree ring width indices were developed (**A**: Tree ring width index; **B**: First-order difference of tree ring width index).

The growth trend of tree width is removed using linear fitting. Considering the characteristics of the tree ring width samples, at least six samples are required to establish a reliable chronology (as shown in **Figure 7**, the darker lines represent reliable chronologies, same below). The Increment borers extracted tree ring width index (IW), drilling resistance extracted tree ring width index (DW), and feed resistance extracted tree ring width index (FW) were ultimately obtained.

By comparing and analyzing the tree ring width indices and their first-order differences (**Figures 7A, B**), there were differences in specific values between the indices extracted by the Increment borers and Resistograph. Nevertheless, the tree ring width index extracted by the Resistograph is generally reliable.

#### 3.3.2 Tree ring density index

**Figure 8:**
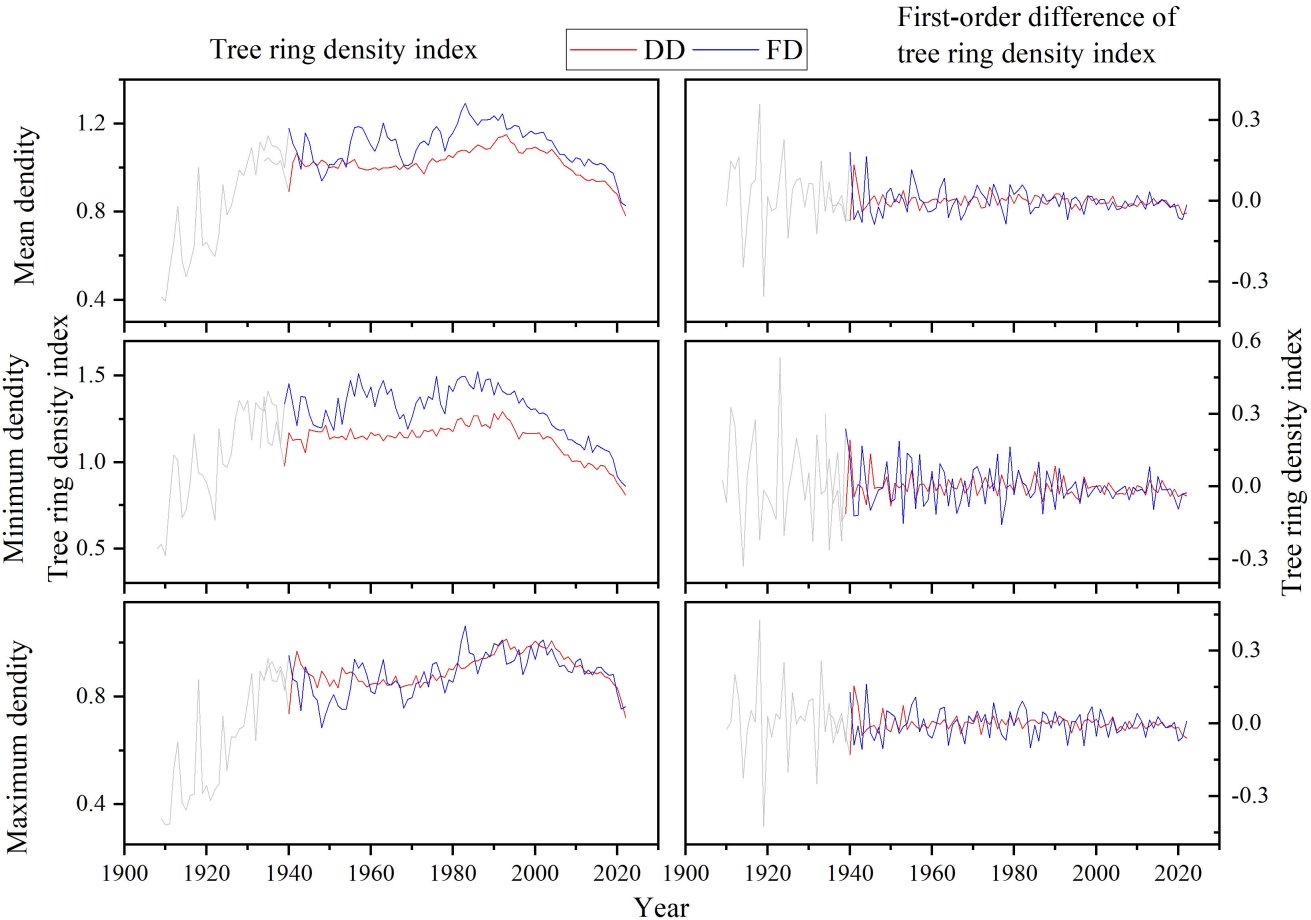
Tree ring density indices of ancient *P.tabulaeformis* at Yuxiang Forest Farm (DD: Tree ring density index extracted by drilling resistance; FD: Tree ring density index extracted by feed resistance).

Using EEMD to remove the residual resistance of the Resistograph and based on the specific characteristics of the sample sequence, at least six samples are required when establishing a balanced chronology (**Figure 8**). The Drilling resistance extracted tree ring mean density (DDmean), maximum density (DDmax), minimum density (DDmin) index, and Feed resistance extracted tree ring mean density (FDmean), maximum density (FDmax), minimum density (FDmin) index for ancient *P.tabulaeformis* at Yuxiang Forest Farm were ultimately obtained.

By comparing the tree ring density indices and their first-order differences extracted by the drilling resistance and feed resistance of the Resistograph, it is found that despite some differences, the tree ring density indices extracted by the Resistograph demonstrate high consistency between drilling and feed resistance methods. The tree ring density index curves extracted by drilling resistance and feed resistance showed higher reliability in both high-frequency and low-frequency signals.

### 3.4 Relationship between tree ring index and climatic factors

**Figure 9:**
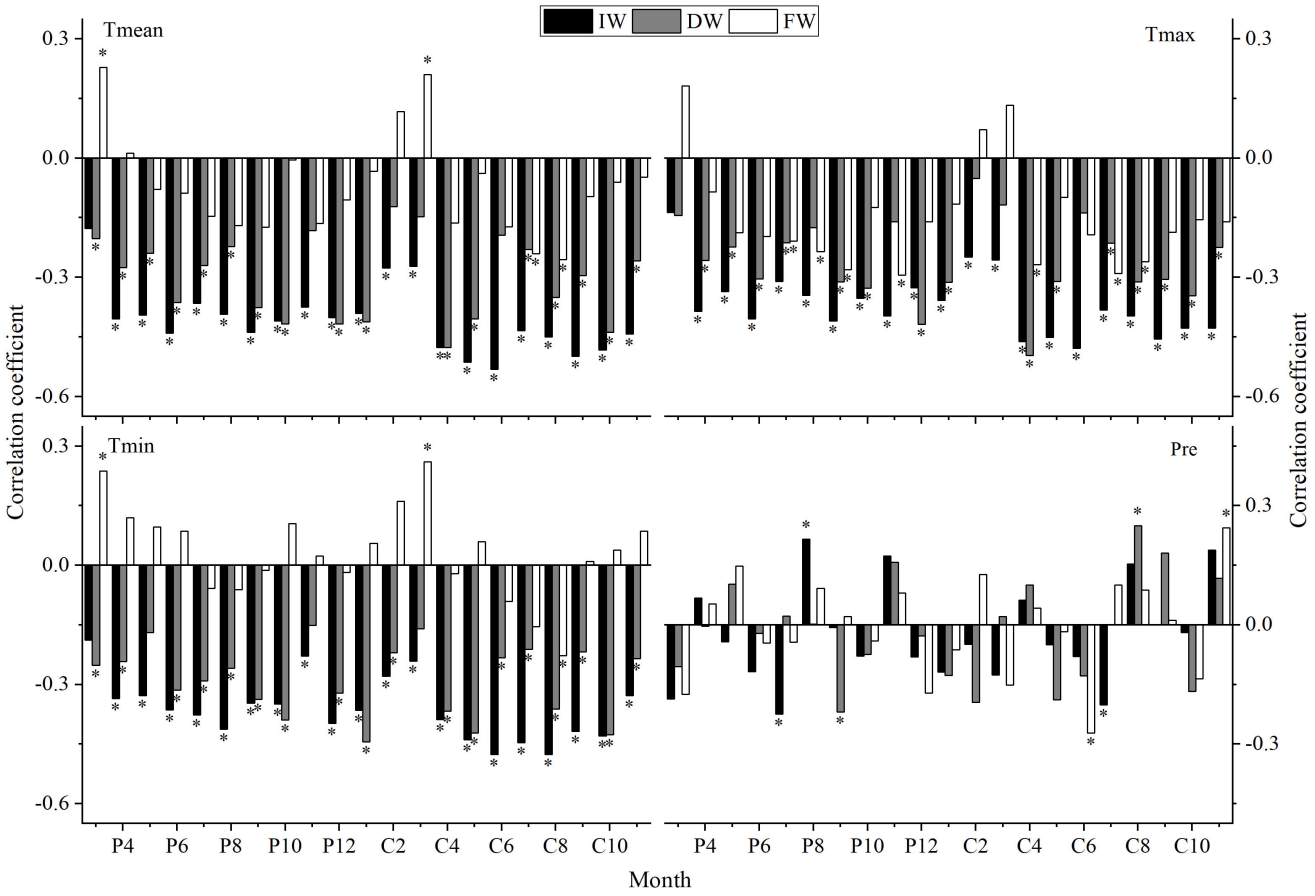
Correlation analyses between different width indices and climatic factors (P: represents the previous year, C: represents the current year; * indicates significant correlation at the 95% confidence level, same below).

**Figure 10:**
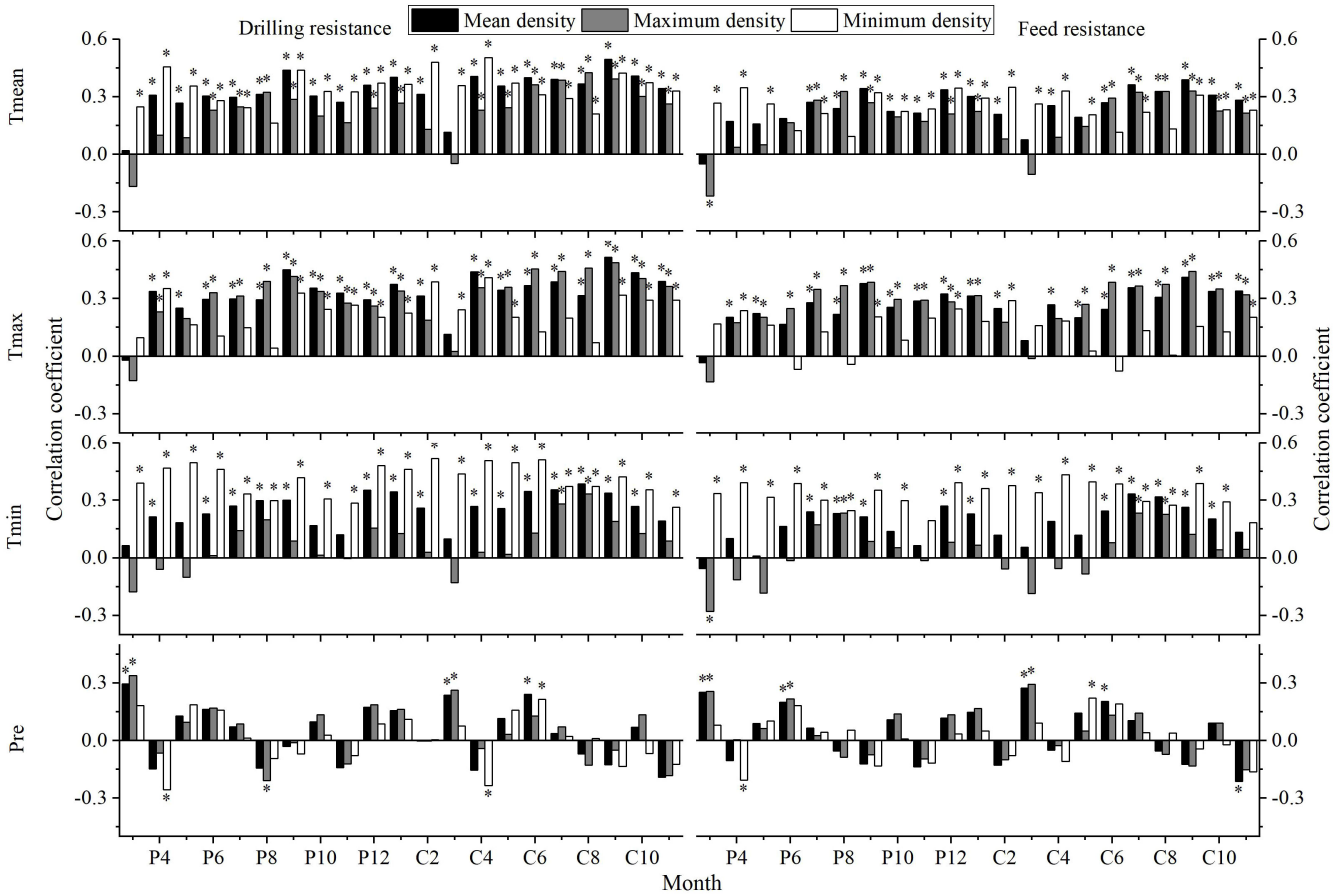
Correlation analyses between different tree ring density indices and climatic factors (P: represents the previous year, C: represents the current year; * indicates significant correlation at the 95% confidence level).

The results of correlation analyses between the IW, DW, FW, DDmean, DDmax, DDmin, FDmean, FDmax, FDmin and the Tmean, Tmax, Tmin, and Pre are shown in **Figures 9**, **10**:

IW and DW both have a high negative correlated with Tmean, Tmax and Tmin from the previous year to the current year; FW is higher negatively correlated with Tmean of C7-8, Tmax of P7-9, P11, C4, C7-8, Tmin of C8; and higher positively correlated with Tmean, Tmin of P3, C3. IW, DW and FW show a weak response to Pre and exhibit significant differences. DW and IW show a high consistency in their response to climate, while there are certain differences in the response of FW to climate compared to IW.

DDmean, DDmax, DDmin, FDmean, FDmax and FDmin exhibit a high consistency in their response to climate, they all show a positive correlation with temperature, they have a weak response to Pre. However, there are certain differences; DDmean, DDmax, and DDmin are more sensitive to climate responses than FDmean, FDmax, and FDmin. Moreover, DDmax and FDmax are more sensitive to responses to Tmax, while DDmin and FDmin are more sensitive to responses to Tmin.

## 4 Discussion

### 4.1 The feasibility of extracting tree-ring information using the Resistograph

In this study, we have innovatively improved upon the traditional automatic tree-ring information extraction methods used by the Resistograph, introducing a comprehensive linear threshold for the extraction of tree-ring information. After validation through correlation analysis, the new method has achieved significant enhancements in extraction accuracy and data reliability, demonstrating higher precision and reliability compared to the original technique.

Through correlation analysis and T-tests between IW, DW, and FW, it was found that DW and FW are significantly correlated with IW (0.41, 0.19, p< 0.01), and the differences are not significant (ρ = 0.55, 0.99). Additionally, the IW, DW, and FW indices exhibit a high level of consistency in their response to climate, mainly influenced by the temperatures of the previous and current years. This means that the tree ring index extracted by the Resistograph using the optimal comprehensive threshold is credible and can be used for studying the tree growth history. However, there are certain differences between DW, FW, and IW, which may be due to the different measurement methods used. When measuring tree ring width, the Increment borers typically identifies the edge of latewood as the ring boundary, whereas the Resistograph identifies the location of maximum resistance rather than the late wood boundary. Furthermore, the correlation coefficient between DW and IW was higher than that between FW and IW, indicating that tree ring width index extracted by the Resistograph using drilling resistance was more accurate than that extracted using feed resistance. This may be due to the lower temporal resolution of feed resistance measurement, which cannot provide enough details to accurately distinguish the growth period of each year, and thus may not be as effective in extracting ring information as drilling resistance. Therefore, drilling resistance is usually a more reliable choice for precise analysis of tree growth history and environmental response (Li et al., 2016). Despite these differences, comparative analysis indicates that the tree-ring width data extracted by the Resistograph remains reliable, particularly the tree-ring information extracted using drilling resistance. This finding holds significant implications for future research on the Resistograph.

### 4.2 Relationship between tree ring and climatic factors

Yuxiang Forest Farm, situated in the low-altitude plain area at an elevation of only 62 meters, is located at the southern edge of ancient *P.tabulaeformis*’s distribution area, where higher temperatures are detrimental to its growth. Higher temperatures may trigger drought stress, which not only affects the photosynthesis and respiration of trees, but may also slows down the rate of cell division. These changes ultimately affect the number of wood cells and the content of cell wall substances, thereby significantly impacting the width and density of tree rings (Xu et al., 2011; Hansen et al., 1997; Zhang et al., 2021). This phenomenon has been widely verified in studies of tree growth (Ruxianguli et al., 2023). The temperature of the previous year has an obvious “lag effect” on the growth of ancient *P.tabulaeformis* (Fritts, 1976; Wu, 1990; Wei, 2018), and excessively high temperatures have an adverse effect on the growth of the current year. During the growing season, high temperatures cause trees to close their stomata and experience “carbon starvation”, reducing photosynthesis and increasing the consumption of respiration, slowing cell division, accelerating lignification, thereby inhibiting radial growth (Li et al., 2022). This aligns with findings from other regions in Henan Province (Peng et al., 2014; 2018; 2019; Zhao et al., 2019) and increasing the density of tree rings (Ruxianguli et al., 2023), this effect has also been reflected in the *Picea crassifolia* Kom of Qilian Mountain (Xu et al., 2011) and *Picea schrenkiana Fischet* Mey in Kongnaes region (Zhang et al., 2011). By the end of the growing season, trees have largely completed cell growth and enter a phase of photosynthetic accumulation. At this point, high temperatures may lead to a reduction in the water supply available to trees or a decrease in water utilization efficiency, which in turn triggers the closure of leaf stomata, reducing cell growth and the demand for photosynthesis (Yang et al., 2021) and thus limit tree growth (Rahman et al., 2016; Zhang et al., 2020). As a result, trees accumulate more photosynthetic products, mainly used to strengthen the thickness of the cell walls of the latewood cells of the tree rings (Petit and Crivellaro, 2014). At this stage, the latewood cells of the trees have a smaller diameter and thicker cell walls (Li et al., 2015), mainly playing a supporting role (Hervé et al., 2004; Sperry et al., 2006), contributing less to the transport of nutrients and water by the tree, with a very low degree of cell hollowing, thus making the density of latewood relatively large. This phenomenon has also been recorded in the *Pinus koraiensis* and *Abies fabri (Mast.) Craib* studies of Changbai Mountain (Ruxianguli et al., 2023).

However, there are certain differences in climatic response of different density indices; the maximum density is more significantly affected by Tmax, while it shows a significant correlation with Tmin only within two to three months prior. This is because Tmax usually occurs during the day, which is the period when the tree’s metabolic activities are most active. When the temperature is too high, it will intensify the transpiration of the tree, leading to drought stress, inhibiting tree growth, causing the annual ring density to begin the growth phase earlier, thus affecting the maximum density of the annual ring (Chen et al., 2010). On the other hand, the annual minimum ring density is more influenced by Tmin, which usually occurs at night and plays a key role in the accumulation of cell wall substances and the lignification process, thereby affecting the value of the annual ring density. Therefore, the intensity of the tree’s photosynthesis and respiration is crucial for the variation in annual ring density (Antonova and Stasova, 1993; Gou et al., 2007; Yoshihiro et al., 2002; Ruxianguli et al., 2023). Nighttime warming will promote leaf respiration and root respiration, stimulating the consumption of carbohydrates and other nutrients within the cells, leading to a reduction in the accumulation of nutrients (Zhu and Zheng, 2022). At this time, the earlywood cells expand more slowly, the cell walls are thicker, the cells have a higher saturation level, and it is easier to form a higher annual ring density.

## 5 Conclusions

This study focuses on *P. tabulaeformis* the Yuxiang Forest Farm in Henan Province, utilizing non-destructive tree ring data collection methods such as the Resistograph and Increment borers to explore the feasibility of extracting tree-ring information using the Resistograph and to discuss the impact of meteorological factors on tree-ring characteristics. The main conclusions drawn are:

(1) The Resistograph’s use of a linear threshold for extracting tree-ring information is more accurate and credible compared to the use of a fixed threshold.
(2) In the Yuxiang Forest Farm, the initial comprehensive threshold for extracting tree-ring information from ancient *P. tabulaeformis* using drilling and feed resistance is 0, with the maximum thresholds being 0.47 and 1.91, respectively. Linear threshold is identified as the optimal comprehensive threshold, which provides the best filtering effect. The tree-ring information extracted is reliable. Moreover, the drilling resistance is more accurate than the feed resistance in extracting tree ring information.
(3) The variations in both width and density of the ancient *P. tabulaeformis* at Yuxiang Forest Farm are primarily influenced by temperature. The maximum density is more significantly affected by the average maximum temperature, whereas the minimum density is more noticeably influenced by the average minimum temperature.

## Data Availability

The original contributions presented in the study are included in the article/supplementary material. Further inquiries can be directed to the corresponding authors.
